# Localization of relaxin‐like gonad‐stimulating peptide expression in starfish reveals the gonoducts as a source for its role as a regulator of spawning

**DOI:** 10.1002/cne.25496

**Published:** 2023-05-22

**Authors:** Yuling Feng, Victor M. Piñon Gonzalez, Ming Lin, Michaela Egertová, Masatoshi Mita, Maurice R. Elphick

**Affiliations:** ^1^ School of Biological & Behavioural Sciences Queen Mary University of London London UK; ^2^ Department of Biochemistry Showa University School of Medicine Tokyo Japan

**Keywords:** echinoderm, gonadotropin, neuropeptide, relaxin, spawning, starfish

## Abstract

Oocyte maturation and gamete release (spawning) in starfish are triggered by relaxin‐like gonad‐stimulating peptide (RGP), a neuropeptide that was first isolated from the radial nerve cords of these animals. Hitherto, it has generally been assumed that the radial nerve cords are the source of RGP that triggers spawning physiologically. To investigate other sources of RGP, here we report the first comprehensive anatomical analysis of its expression, using both in situ hybridization and immunohistochemistry to map RGP precursor transcripts and RGP, respectively, in the starfish *Asterias rubens*. Cells expressing RGP precursor transcripts were revealed in the ectoneural epithelium of the radial nerve cords and circumoral nerve ring, arm tips, tube feet, cardiac stomach, pyloric stomach, and, most notably, gonoducts. Using specific antibodies to *A. rubens* RGP, immunostaining was revealed in cells and/or fibers in the ectoneural region of the radial nerve cords and circumoral nerve ring, tube feet, terminal tentacle and other arm tip‐associated structures, body wall, peristomial membrane, esophagus, cardiac stomach, pyloric stomach, pyloric caeca, and gonoducts. Our discovery that RGP is expressed in the gonoducts of *A. rubens* proximal to its gonadotropic site of action in the gonads is important because it provides a new perspective on how RGP may act as a gonadotropin in starfish. Thus, we hypothesize that it is the release of RGP from the gonoducts that triggers gamete maturation and spawning in starfish, while RGP produced in other parts of the body may regulate other physiological/behavioral processes.

## Graphical abstract

Oocyte maturation and release (spawning) in starfish are triggered by relaxin‐like gonad‐stimulating peptide (RGP), which exerts its effects by stimulating ovarian follicle cells to produce 1‐methyladenine. Here, we present evidence that gonoducts (Gd) located proximal to the ovaries (O) are the physiological source of RGP that triggers spawning in starfish.

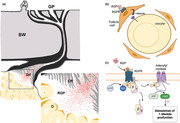

## 1 INTRODUCTION

Reproduction in starfish (phylum Echinodermata), as in many other marine invertebrates, is enabled by broadcast spawning—the release of millions of gametes (eggs and sperm) into the surrounding sea water, which is then followed by external fertilization. Because gamete production is energetically costly, it is important that the timing of spawning by different individuals within a population is synchronized. Accordingly, there is evidence that a combination of external stimuli triggers spawning, including changes in day length, the lunar cycle, water temperature, and pheromones released by conspecifics (Byrne et al., 1997; Lawrence, 2013; Pearse & Walker, 1986; Pearse et al., 1986). Sensory detection of these external stimuli then leads to activation of internal endocrine and/or neural gonadotropic mechanisms that regulate gamete maturation and expulsion of gametes from gonads into the surrounding seawater.

Evidence of the existence of a gonadotropic neurohormone in starfish was first reported in 1959 with the discovery that extracts of radial nerve cords from the starfish *Asterias forbesi* induce spawning when injected into the coelom of reproductively mature male or female starfish (Chaet & McConnaughy, 1959). Subsequent efforts to determine the molecular identity of the active component of radial nerve cord extracts revealed it to be a peptide, which became known as gonad‐stimulating substance or gamete‐shedding substance (GSS). Furthermore, purification of GSS from several starfish species enabled determination of its amino acid composition and estimation of its molecular mass (Chaet, 1966a, 1966b; Kanatani et al., 1971). However, it was not until 2009 that the molecular structure of GSS was finally determined (Mita, Yoshikuni, et al., 2009). GSS was purified from extracts of radial nerve cords from the starfish *Patiria pectinifera* and found to be a heterodimer comprising an A‐chain and a B‐chain. These two chains are linked by three disulfide bridges, with two interchain bridges between the A‐chain and B‐chain, and an intrachain bridge within the A chain. Furthermore, sequencing revealed that the A‐chain and B‐chain have unique cysteine motifs that are a feature of the insulin/insulin‐like growth factor (IGF)/relaxin superfamily, and phylogenetic analysis revealed that GSS is more closely related to the relaxin subfamily than the insulin/IGF subfamily. Therefore, GSS was classified as an invertebrate member of the insulin/IGF/relaxin superfamily and was renamed as relaxin‐like gonad‐stimulating peptide (RGP) (Mita, Yoshikuni, et al., 2009). RGP has subsequently been identified in other starfish species, including *Asterias amurensis*, (Mita, Daiya, et al., 2015), *Acanthaster planci* cf. *A. solaris* (Mita, Ikeda, et al., 2015; Smith et al., 2019), *Aphelasterias japonica* (Mita & Katayama, 2016), *Asterias rubens* (Lin, Mita, et al., 2017; Semmens et al., 2016), and *Astropecten scoparius* (Mita, Osugi, et al., 2021), revealing evolutionary conservation of both its structure and function. Relaxin‐type peptide precursors have also been identified in other echinoderms, including ophiuroids (brittle stars), holothurians (sea cucumbers), and crinoids (feather stars) (Aleotti et al., 2022; Chieu et al., 2019; Suwansa‐Ard et al., 2018; Zandawala et al., 2017). Furthermore, a relaxin‐type peptide triggers spawning in the sea cucumber *Holothuria scabra*, showing that the gonadotropic action of relaxin‐type peptides also extends to other echinoderms (Chieu et al., 2019).

Prior to the molecular identification of RGP in starfish, its mechanism of action as a regulator of oocyte maturation and spawning was investigated. This revealed that it binds to receptors expressed by follicle cells that surround oocytes and stimulates production of 1‐methyladenine, which then triggers germinal vesicle breakdown and oocyte maturation (Hirai & Kanatani, 1971; Kanatani et al., 1969; Mita, 2019). RGP exerts its effects via G‐protein‐dependent cyclic adenosine monophosphate (cAMP) synthesis in follicle cells (Mita et al., 1989, 2011, 2012), but the biochemical pathway by which this leads to production of 1‐methyladenine is not known (Mita, 2019).

Although GSS/RGP was first isolated from starfish radial nerve cords, use of a variety of experimental techniques has revealed that this gonadotropic peptide is also present in other tissues/organs in starfish, albeit at lower concentrations than in the radial nerve cords. Thus, use of a bioassay for gonadotropic activity revealed the presence of low concentrations of GSS in tube feet, body wall, and cardiac stomach in *A. amurensis* and *P. pectinifera* (Kanatani & Ohguri, 1966). A more recent bioassay analysis of extracts of *P. pectinifera* tissues/organs revealed high concentrations of GSS activity in radial nerves and circumoral nerve rings and low concentrations of GSS activity in cardiac stomach and tube feet, but no GSS activity was detected in pyloric caeca, ovaries, or testes (Mita, Ito, et al., 2009). With the identification of RGP, quantification of transcript abundance in various tissues by qPCR revealed high levels in radial nerve cords, lower levels in cardiac stomach and pyloric caeca, and trace levels in tube feet, ovaries, or testes (Haraguchi et al., 2016). More recently, specific antibodies to RGP have been generated (Katayama & Mita, 2016) and used for immunoassays, enabling quantification of RGP in the radial nerve cords and circumoral nerve ring of *P. pectinifera*. However, using immunoassays RGP was not detected in cardiac stomach, pyloric stomach, pyloric caeca, tube feet, ovaries, or testes in *P. pectinifera* (Mita & Katayama, 2018; Yamamoto et al., 2017). Transcriptomic and proteomic analysis of a variety of tissues/organs from the crown‐of‐thorns starfish *A. planci* cf. *A. solaris* has revealed the presence of RGP precursor transcripts and/or RGP in the radial nerve cords, tube feet, terminal tentacle, body wall spines, and gonads. Furthermore, it is noteworthy that RGP precursor transcripts were also detected in coelomocytes (Jonsson et al., 2022; Smith et al., 2017).

More specific localization of RGP expression in starfish at a cellular level has been examined using mRNA in situ hybridization. This was initially restricted to analysis of the radial nerve cords, revealing a small population of cells located in the ectoneural epithelial layer in *P. pectinifera* (Mita, Yoshikuni, et al., 2009). Subsequently, a more comprehensive analysis of RGP expression was performed by applying mRNA in situ hybridization in the starfish *A. rubens*. Consistent with findings from *P. pectinifera*, cells expressing RGP transcripts were revealed in the ectoneural epithelium of the radial nerve cords of *A. rubens*. In addition, cells expressing RGP transcripts were revealed in the circumoral nerve ring and tube feet (Lin, Mita, et al., 2017). Furthermore, an extensive population of cells expressing RGP transcripts was revealed in the arm tips of *A. rubens*, largely concentrated in the body wall external epithelium surrounding the sensory terminal tentacle and the associated optic cushion (simple eye). Informed by this new insight into the anatomy of RGP expression in starfish, it was proposed that the arm tips, and not the radial nerve cords, may be the physiological source of RGP that triggers spawning in starfish (Lin, Mita, et al., 2017). The rationale for this hypothesis was that RGP‐expressing cells in the arm tip being located proximal to the terminal tentacle and associated sensory organs are ideally positioned to integrate sensation of changes in environmental conditions (e.g., day length, lunar cycle, pheromones) thought to trigger spawning. However, it is not known if the RGP‐expressing cells in the arm tips have axonal processes that terminate at sites whereby RGP released by these cells could gain access to the coelomic cavity where the gonads are located. To address this question, immunohistochemical methods need to be employed and this has recently become feasible with development of specific antibodies to RGP (Katayama & Mita, 2016; Yamamoto et al., 2017). Consistent with patterns of RGP expression revealed by in situ hybridization, use of immunohistochemical methods has revealed RGP‐immunoreactive cells and processes in the ectoneural region of the radial nerve cords in *P. pectinifera* (Yamamoto et al., 2017). However, a more extensive analysis of RGP expression in starfish using immunohistochemical methods has yet to be reported. Here, we have combined use of both mRNA in situ hybridization and immunohistochemistry to investigate comprehensively the anatomical expression pattern of RGP in the starfish *A. rubens*. This has been facilitated by generation of specific antibodies to *Asterias* RGP, which has an identical structure in *A. amurensis* and *A. rubens* (Katayama et al., 2019; Mita, Elphick, et al., 2021).

## 2 MATERIALS AND METHODS

### 2.1 Animals

Specimens of *A. rubens* were obtained from a fisherman based at Whitstable (Kent, UK) and then transported to and maintained in an aquarium with recirculating artificial seawater under a 12‐h light–dark cycle (lights on at 8:00 a.m.) at 12°C in the School of Biological & Behavioural Sciences at Queen Mary University of London. Starfish were fed ad libitum on mussels (*Mytilus edulis*). After collection of starfish, animals used here for analysis of RGP expression were fixed immediately or only kept in the aquarium for less than a week prior to fixation.

### 2.2 Localization of AruRGPP transcripts in *A. rubens* using in situ hybridization

Digoxigenin‐labeled RNA antisense probes complementary to AruRGP precursor (AruRGPP; GenBank accession numbers KT601728 and ALJ99970) transcripts and corresponding sense probes were synthesized, as reported previously (Lin, Mita, et al., 2017). Because a second relaxin‐type peptide precursor (ArRLPP2) has been identified in *A. rubens* (Semmens et al., 2016), we compared the sequences of the AruRGPP and ArRLPP2 transcripts to assess potential hybridization of the AruRGPP antisense probes with ArRLPP2 transcripts, and this revealed only 52% nucleotide sequence identity in the coding regions.

To investigate AruRGP expression throughout the body, small specimens (∼4 cm in diameter) were analyzed. Starfish of this size were used so that sections of arms or central disks could be collected on microscope slides, but it was not possible to determine the gender of these animals. However, to investigate AruRGP expression in the reproductive system, V‐shaped regions of the body at the junction between adjacent arms that incorporated a pair of gonoducts and gonads were dissected from larger animals (∼15 cm in diameter; one male and two females). Whole starfish or body regions were then fixed in 4% paraformaldehyde (PFA; Sigma–Aldrich, Gillingham, UK) for 2 days at 4°C and then whole starfish were dissected to separate arms from the central disc region. After decalcification with Morse's solution (10% sodium citrate and 20% formic acid in autoclaved water) for 8 h (replenished every 2 h), the decalcified body parts were washed in autoclaved water and dehydrated through an ethanol series (50%, 70%, 90%, and 100%). Body parts were cleared in xylene in two steps (5 min followed by 8 min) and then embedded in molten paraffin wax. Sectioning of blocks at 13 μm thickness was performed using a microtome (RM2145, Leica Microsystems, Milton Keynes, UK) and then sections were mounted on SuperFrost Plus microscope slides (VWR, Lutterworth, UK).

Slides were placed in an oven at 60°C for 1 h and then xylene was used to remove wax, followed by rehydration through a descending ethanol series (100%, 90%, 70%, 50%, and 30%; 7 min in each step). After washing in phosphate‐buffered saline (PBS; pH 7.3; prepared using disodium phosphate, monosodium phosphate, and sodium chloride purchased from VWR) for 3 × 5 min and postfixation in 4% PFA/PBS for 20 min, slides were incubated with proteinase K (Novagen, an Affiliate of Merck KGaA, Darmstadt, Germany) solution at a concentration of 10 μg/mL in a buffer containing 50 mM Tris‐HCl and 6.25 mM EDTA for 20 min at 37°C. After washing in PBS, slides were acetylated for 10 min in 1.325% triethanolamine, 0.25% acetic anhydride, and 0.175% acetic acid (VWR) made up in distilled water and mixed well with stirring. Slides were then washed in PBS and 5× saline sodium citrate (SSC; prepared using sodium chloride and sodium citrate purchased from VWR) buffer (1 × 5 min) at room temperature. Before performing probe hybridization, slides were prehybridized in hybridization buffer (50% formamide; 5× SSC; 500 μg/mL yeast total RNA; 50 μg/mL heparin; 0.1% Tween‐20 in distilled water) in a humid chamber for 2 h at room temperature. A total of 1000 ng/mL DIG‐labeled antisense or sense RNA probes made up in hybridization buffer were denatured by heating at 80°C for 2 min and then were applied to slides (150 μL/slide). Slides were then covered with parafilm and incubated for 48 h at 50°C.

Next, slides were placed in warm 5× SSC to remove the parafilm and then washed in 0.2× SSC (2 × 40 min at 50°C; 1 × 10 min at room temperature) followed by a wash in buffer B1 (10 mM Tris‐HCl, pH 7.5; 150 mM NaCl in autoclaved water) for 10 min at room temperature. Then slides were blocked with 5% goat serum (Sigma–Aldrich) diluted in B1 buffer in a humid chamber for 2 h at room temperature. After the blocking step, slides were incubated with alkaline phosphatase‐conjugated anti‐DIG antibody (Roche Diagnostics GmbH, Mannheim, Germany) at 1:2000 dilution in 2.5% goat serum/B1 buffer in a humid chamber overnight at 4°C.

Next, slides were washed in B1 buffer (3 × 5 min) followed by buffer B3 (100 mM Tris‐HCl, pH 9.5; 100 mM NaCl; 50 mM MgCl_2_ in distilled water) for 10 min at room temperature. Alkaline phosphatase substrate was prepared in buffer B3 by adding 4.5 μL/mL of nitro blue tetrazolium chloride (NBT; Sigma–Aldrich) stock solution (75 mg/mL) in 70% dimethylformamide (Sigma–Aldrich) and 3.5 μL/mL of 5‐bromo‐4‐chloro‐3′‐indolyphosphate p‐toluidine (BCIP; ThermoFisher Scientific, Dartford, UK) stock solution (50 mg/mL in 70% dimethylformamide) and applied to slides (500 μL/slide), incubating overnight at 4°C until staining was observed. Slides were washed in distilled water to terminate the reaction (3 × 5 min) and then slides were dried on a hotplate and placed in 100% ethanol (2 × 10 s). After clearing in xylene (2 × 7 min), slides were mounted with coverslips using HistoMount (National Diagnostics) mounting medium.

### 2.3 Antibody characterization

The generation and characterization of the AruRGP antiserum used here for immunohistochemistry, as described below in Section 2.4, have been reported previously and, using western blotting and an enzyme‐linked immunosorbent assay (ELISA), the specificity and sensitivity of the antibodies to AruRGP were determined (Katayama et al., 2019; Mita, Elphick, et al., 2021). This revealed that the antibodies to AruRGP do not cross‐react with *Patiria pectinifera* RGP (PpeRGP), which shares 64% amino acid identity with AruRGP. By way of comparison, a second relaxin‐type peptide (ArRLP2) that has been identified in *A. rubens* (Semmens et al., 2016) shares only 37% amino acid identity with AruRGP. Furthermore, here the specificity of immunostaining observed with the AruRGP antiserum was assessed by performing preabsorption experiments. The AruRGP antiserum (1:1600 in PBS) was incubated with AruRGP (20 μM; synthesized by the Peptide Institute, Osaka, Japan) on a rocking table at room temperature for 2 h. After dilution of the preabsorbed antiserum to 1:16,000 with 5% normal goat serum/PBS containing 0.1% Tween‐20 (PBST), preabsorbed antiserum was tested on sections of starfish arms or disks, as described below, with adjacent sections incubated with the AruRGP antiserum at the same dilution (1:16,000). Assessment of the specificity of the AruRGP antiserum was also achieved by comparison of staining patterns observed using immunohistochemistry and staining patterns observed using in situ hybridization for localization of AruRGP precursor transcripts. The AruRGP antiserum has been assigned the RRID number RRID:AB_2936916.

### 2.4 Localization of AruRGP expression using immunohistochemistry

To investigate AruRGP expression in *A. rubens* using immunohistochemistry, animals of varying size and maturity were analyzed. For general investigation of expression throughout the starfish body, whole juvenile starfish (∼1 cm in diameter) and arms and the central disc from specimens of *A. rubens* with a diameter of ∼4 cm were analyzed. Starfish of this size were used so that sections of arms or central disks could be collected on microscope slides, but it was not possible to determine the gender of these animals. However, to enable analysis of expression in the reproductive system, V‐shaped regions of the body at the junction between adjacent arms that incorporated a pair of gonoducts and gonads were dissected from larger animals (∼15 cm in diameter; two male and two female specimens).

Whole starfish or regions of the body were fixed in Bouin's solution (75 mL saturated picric acid in seawater, 25 mL 37% formaldehyde, 5 mL acetic acid) at 4°C for 3 days. Following decalcification in 4% ascorbic acid and 0.3 M sodium chloride (1:1 solution) for 2 weeks at 4°C with regular changes, whole starfish or body parts were embedded in paraffin wax, sectioned at 10 μm using a microtome (RM2145, Leica Microsystems, Milton Keynes, UK), and mounted on chrome alum–gelatin‐coated glass slides. Wax was removed from sections using xylene (3 × 10 min at room temperature) and slides were placed in 100% ethanol (2 × 10 min). After incubating slides in 1% hydrogen peroxide in methanol for 30 min to quench endogenous peroxidase, slides were rehydrated through a graded series of ethanol (90%, 70%, and 50%; 10 min for each step) into distilled water. The slides were washed once in PBS, once in PBST, and then were incubated with 5% normal goat serum (Sigma–Aldrich; diluted in PBST) for 2 h to block nonspecific antibody‐binding sites in tissue sections. Slides were then incubated overnight with the rabbit AruRGP antiserum, which was used at a dilution of 1:16,000 (whole arm or central disk sections) or 1:2000 (body region containing reproductive organs). After washing slides with PBST (5 × 10 min), slides were incubated with secondary antibodies (goat anti‐rabbit horseradish peroxidase conjugated immunoglobulins [Jackson ImmunoResearch via Stratech Scientific, Newmarket, Suffolk, UK] diluted 1:500 in 2% normal goat serum/PBST) for 3 h. Immunostaining was visualized using diaminobenzidine (VWR) and after intense immunostaining was observed, the reaction was terminated by washing in distilled water (2 × 10 min). Following dehydration through an ethanol series (50%, 70%, 90%, and 2 × 100%; 10 min each), slides were cleared in xylene (2 × 10 min) and mounted with coverslips using DPX Mountant (VWR).

### 2.5 Imaging

An Infinity Analyse Camera (Teledyne Lumenera INFINITY5‐5C) attached to a Leica DMRA2 light microscope and Infinity Analysis 7 software running on an iMac computer (27‐inch with OS X Yosemite, v. 10.10) were used to capture photographs of sections. Scale bars in images were added using ImageJ Fiji (Schindelin et al., 2012) and images were compiled into montages and labeled using Adobe Photoshop CC2020 (San Jose, CA).

### 2.6 In vitro pharmacology

Informed by analysis of the expression of AruRGP precursor transcripts and AruRGP in *A. rubens*, AruRGP (synthesized by the Peptide Institute) was tested for myoactivity on cardiac stomach (*n* = 3) and tube foot (*n* = 3) preparations. The methodology employed for in vitro testing of the effects of neuropeptides on cardiac stomach and tube foot preparations has been reported previously (Elphick et al., 1995; Lin, Egertova, et al., 2017; Melarange & Elphick, 2003; Tinoco et al., 2021, 2018; Zhang et al., 2020, 2022). Here, AruRGP was tested at a concentration of 1 μM for myoexcitatory (contraction) and myoinhibitory (relaxation) effects. To facilitate detection of potential relaxing effects, preparations were precontracted with 30 mM KCl (cardiac stomach) or 10 μM acetylcholine (tube feet). The SALMFamide neuropeptide S2 (1 μM; custom synthesized by Peptide Protein Research Ltd, Fareham, UK) was also tested on cardiac stomach preparations as a positive control for myorelaxant activity (Melarange & Elphick, 2003).

## 3 RESULTS

**FIGURE 1:**
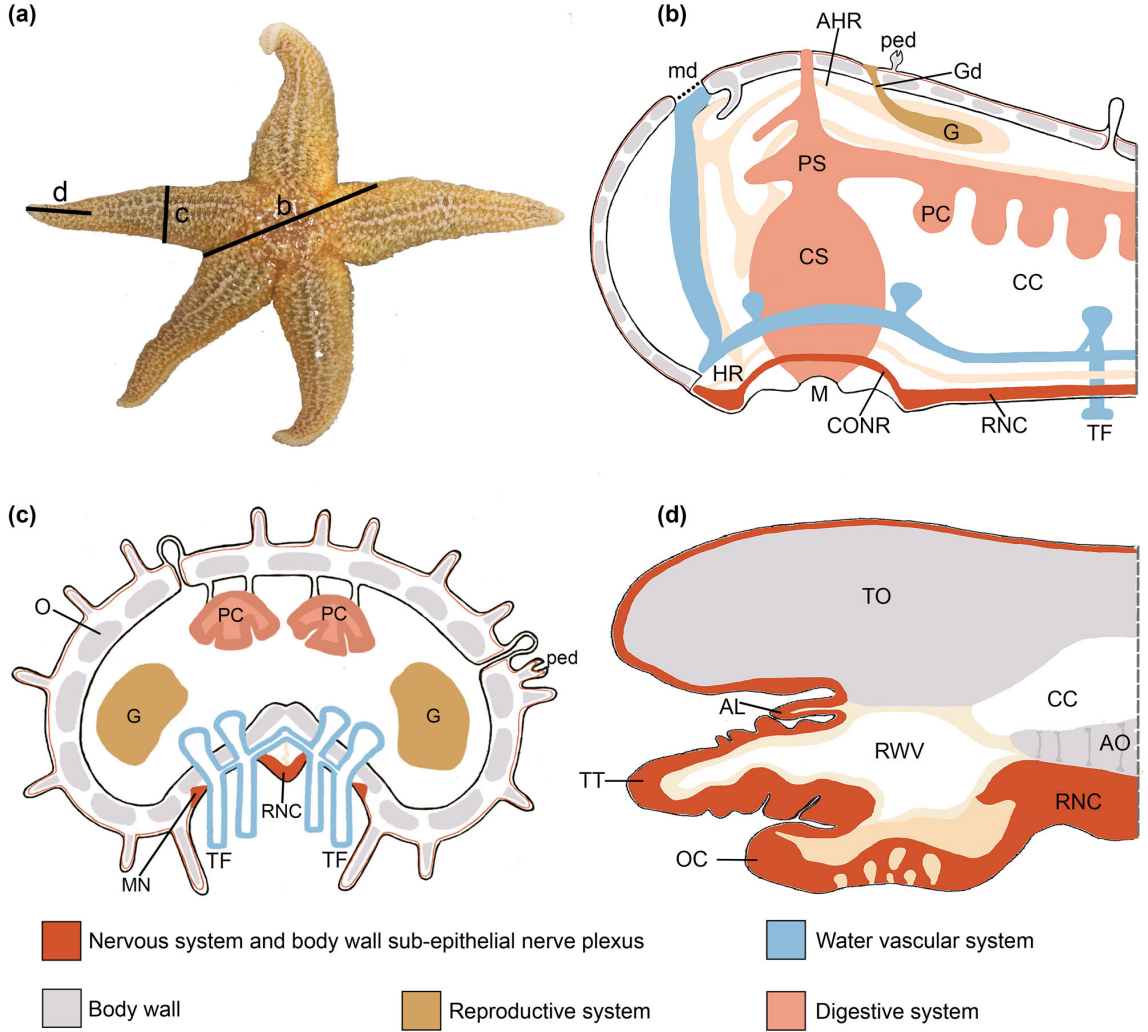
Starfish anatomy. (a) Photograph of a specimen of *Asterias rubens*. The lines labeled b, c, and d show the position and orientation of the diagrams in panels b, c, and d, respectively. (b) Schematic diagram of the central disc and proximal region of an arm. (c) Schematic diagram of a transverse section of an arm. (d) Schematic diagram of a sagittal section through the tip of an arm. AHR, aboral hemal ring; AL, aboral lappet; AO, ambulacral ossicles; CS, cardiac stomach; CONR, circumoral nerve ring; CC, coelomic cavity; G, gonad; Gd, gonoduct; HR, hemal ring; md, madreporite; MN, marginal nerve; O, ossicle; OC, optic cushion; PC, pyloric caecum; PS, pyloric stomach; ped, pedicellaria; RNC, radial nerve cord; RWV, radial water vessel; TF, tube foot; TO, terminal ossicle; TT, terminal tentacle. The photograph in Figure 1a was taken using an iPhone13 pro with a 12‐megapixel sensor camera (Apple, USA) and then background removal and labeling were accomplished using Photoshop CC2020 (San Jose, CA, USA). Images b and c were adapted from Yanez‐Guerra et al. (2018) and image d was adapted from Smith (1937). Images b–d were drawn in Photoshop CC2020 (San Jose, CA) using the digital pen of a creative drawing tablet (Wacom, Tokyo, Japan), which was connected to a laptop (MacBook Pro, Apple, USA).

Expression of AruRGPP and AruRGP in *A. rubens* was revealed using mRNA in situ hybridization and immunohistochemistry, respectively, as described in detail below. To facilitate interpretation of the patterns of staining reported here, the anatomy of starfish is illustrated in Figure 1.

### 3.1 Localization of AruRGP precursor transcripts in *A. rubens* using in situ hybridization

#### 3.1.1 Radial nerve cords, tube feet, and arm tips

**FIGURE 2:**
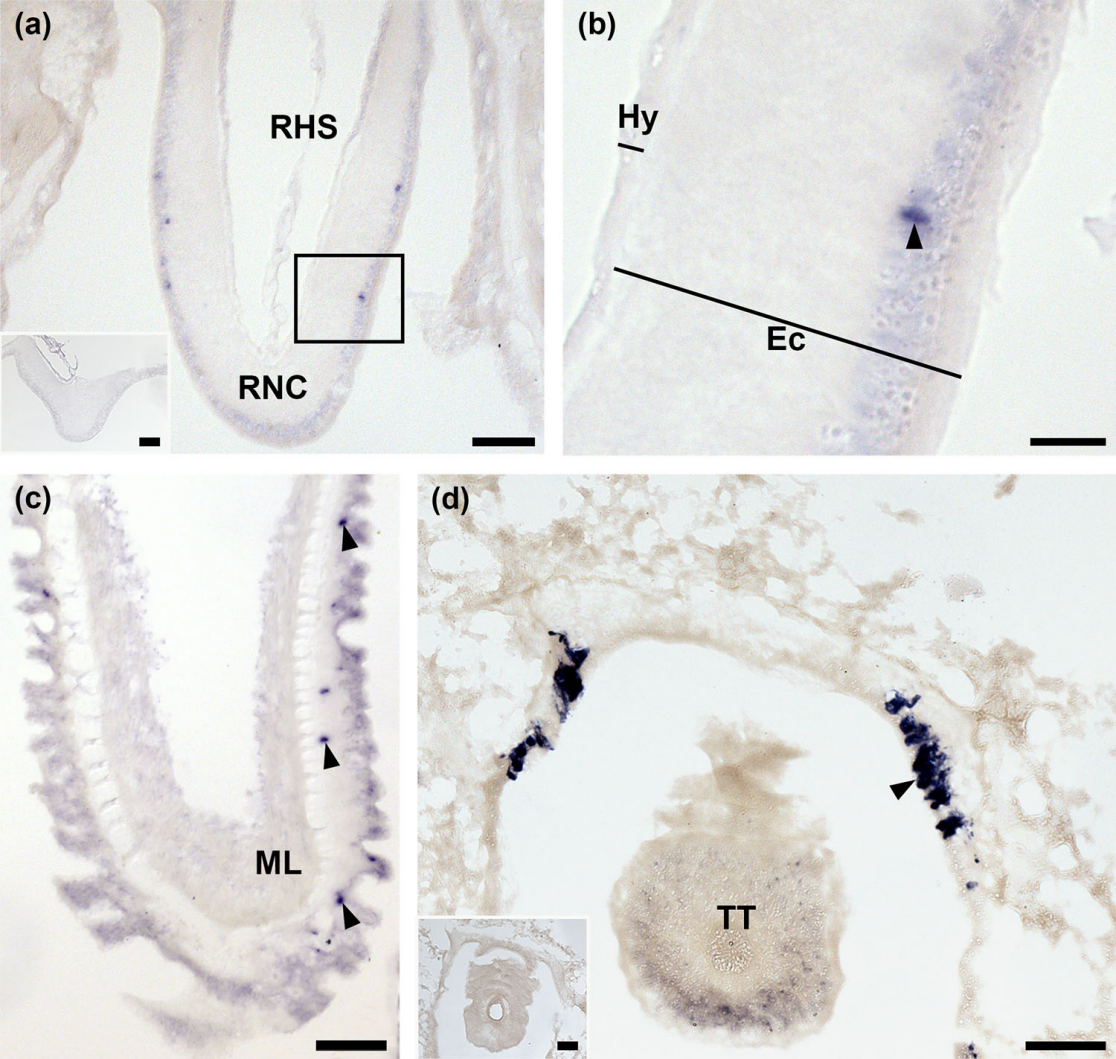
Localization of AruRGP precursor transcripts in radial nerve cord, tube foot, and arm tip of *Asterias rubens* using in situ hybridization. (a) Transverse section of a radial nerve cord incubated with antisense probes, revealing a bilaterally symmetrical pattern of staining, with two or three stained cells located in the epithelium of the ectoneural region on both sides. The inset shows absence of staining in a radial nerve cord incubated with AruRGP precursor sense probes (see also Figure S1a). (b) A higher magnification image of the boxed region in panel a. (c) Longitudinal section of a tube foot showing stained cells (arrowheads) in the subepithelial nerve plexus of the podium. (d) Transverse section of an arm tip showing strongly stained cells (arrowhead) in the body wall epithelium lining the cavity that surrounds the terminal tentacle. The inset of panel d shows absence of staining in a transverse section of an arm tip incubated with sense probes (see also Figure S1b). Ec, ectoneural region; Hy, hyponeural region; RHS, radial hemal strand or sinus; RNC, radial nerve cord; TT, terminal tentacle. Scale bars: 50 μm in panels a, c, and d and in insets of panels a and d; 30 μm in panel b.

Analysis of AruRGP precursor (AruRGPP) expression in *A. rubens* using mRNA in situ hybridization revealed stained cells in the radial nerve cords, tube feet and arm tips, with the specificity of staining confirmed by control experiments using sense probes, where no staining was observed (Figure 2a,d insets; Figure S1a,b). Thus, in transverse sections of arms (refer to Figure 1a,c for anatomy) bilaterally symmetrical groups of two or three AruRGPP‐expressing cells can be observed in the epithelium of the ectoneural region of the radial nerve cords (Figure 2a,b). No AruRGPP‐expressing cells were observed in the hyponeural region of the radial nerve cords (Figure 2a,b). In longitudinal sections of tube feet, cells expressing AruRGPP were observed along the length of the podium, located beneath the external epithelium (Figure 2c). In transverse sections of arm tips (refer to Figure 1a,d for anatomy) AruRGPP expression was revealed in cells located in the body wall epithelium lining the cavity that surrounds the terminal tentacle (Figure 2d).

#### 3.1.2 Digestive system

**FIGURE 3:**
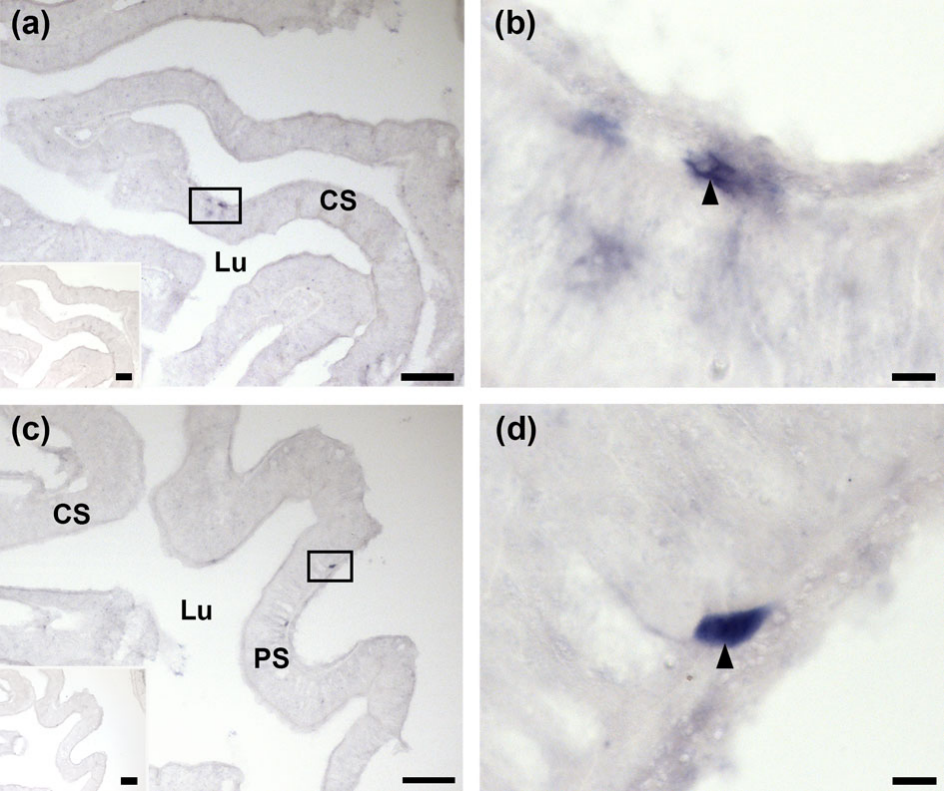
Localization of AruRGP precursor transcripts in the digestive system of *Asterias rubens* using in situ hybridization. (a) Staining (boxed region) in a section of cardiac stomach incubated with AruRGP precursor antisense probes. The inset shows absence of staining in an adjacent section of cardiac stomach incubated with AruRGP precursor sense probes (see also Figure S1c). (b). A higher magnification image of the boxed region in panel a, showing a stained cell (arrowhead). (c) Staining (boxed region) in a section of pyloric stomach incubated with AruRGP precursor antisense probes. The inset shows absence of staining in an adjacent section of pyloric stomach incubated with AruRGP precursor sense probes (see also Figure S1d). (d) A higher magnification image of the boxed region in panel c, showing a stained cell (arrowhead). CS, cardiac stomach; Lu, lumen of gut; PS, pyloric stomach. Scale bar: 100 μm in panel c and in insets of panels a and c; 8 μm in panels b and d.

The digestive system of *A. rubens* includes the highly folded and evertible cardiac stomach, which is linked by a short tubular esophagus to the mouth located on the underside of the central disk. Aboral to the cardiac stomach is the much smaller pyloric stomach, which is linked via pyloric ducts to paired digestive organs (pyloric caeca) located in each arm (Anderson, 1954; Jangoux, 1982) (refer to Figure 1b,c for anatomy). Cells expressing the AruRGPP transcripts were revealed in both the cardiac stomach (Figure 3a,b) and the pyloric stomach (Figure 3c,d) of *A. rubens*, but these cells were sparsely distributed, with only one or two stained cells typically observed in transverse sections of the central disk region. The stained cells are located proximal to the basiepithelial nerve plexus in both the cardiac stomach and pyloric stomach (Figure 3). Furthermore, the specificity of the staining was confirmed by an absence of stained cells in sections of the central disk incubated with sense probes (Figure 3a,c insets; Figure S1c,d). No AruRGPP expression was observed in the pyloric caeca.

#### 3.1.3 Reproductive system

**FIGURE 4:**
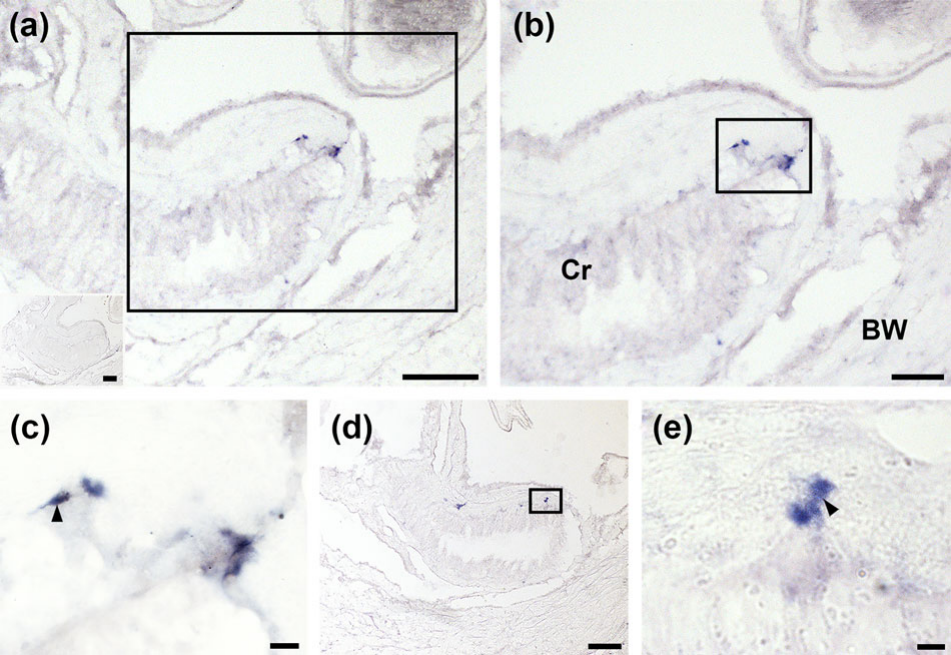
Localization of AruRGP precursor transcripts in the reproductive system of *Asterias rubens* (female) using in situ hybridization. (a) Longitudinal section of a gonoduct incubated with AruRGP antisense probes showing stained cells at the base of the cross‐ridge epithelium, proximal to the junction between the gonoduct and the body wall. The inset shows absence of staining in an adjacent section of gonoduct incubated with AruRGP precursor sense probes (see also Figure S1e). (b) Higher magnification image of boxed region in panel a. (c) Higher magnification image of boxed region in panel b; one of the stained cells (arrowhead) clearly has a stained process emanating from it. (d) Staining in an adjacent section to that shown in panels a–c. (e) Higher magnification image of the boxed region in panel d. BW, body wall; Cr, cross‐ridge epithelium. Scale bar: 100 μm in panels a and d and in inset in panel a; 50 μm in panel b; 8 μm in panels c and e.

The reproductive system of *A. rubens* comprises 10 gonads (ovaries or testes), with a pair of gonads located in each of the five arms. Each gonad is linked via a short gonoduct to the body wall of the arm, proximal to its junction with the central disk (refer to Figure 1b for anatomy). The gonoduct perforates the body wall and opens to the external environment via several gonopores. The luminal lining of the gonoduct comprises an epithelial layer with cross‐ridges, which span the lumen of the duct and imbricate, partially blocking the lumen (Walker, 1974, 1975). No AruRGPP expression was observed in the gonads. However, cells expressing AruRGPP transcripts were revealed in the gonoducts in both female (Figure 4) and male (not shown) animals. Thus, in longitudinal sections of the gonoduct, stained cells can be observed at the base of the cross‐ridges, proximal to the junction of the gonoduct with the body wall (Figure 4a–e). Furthermore, the specificity of this staining was confirmed by an absence of staining in sections of gonoducts incubated with sense probes (Figure 4a, inset; Figure S1e).

### 3.2 Localization of AruRGP expression in *A. rubens* using immunohistochemistry

#### 3.2.1 Central nervous system

**FIGURE 5:**
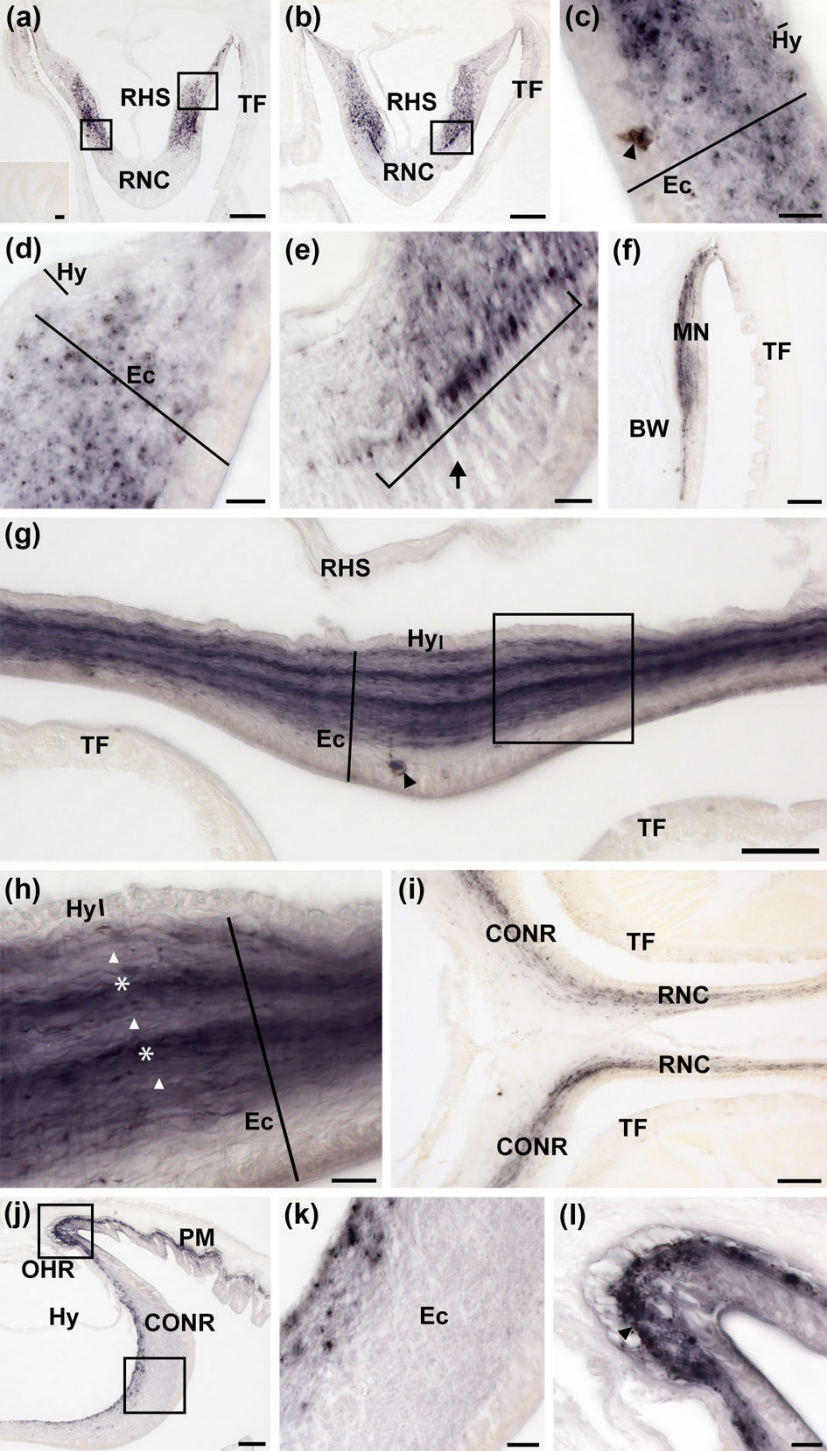
Immunohistochemical localization of AruRGP in the nervous system of *Asterias rubens*. (a, b) AruRGP‐immunoreactivity in transverse sections of radial nerve cord from the regions of an arm proximal to the arm tip (a) or proximal to the circumoral nerve ring (b). The inset of panel a shows absence of immunostaining in a transverse section of the radial nerve cord incubated with AruRGP antiserum that had been preabsorbed with AruRGP (see also Figure S1f). (c) Higher magnification image of the boxed region on the left side of panel a, showing a cluster (two or three) of immunostained cells in the subcuticular epithelium of the ectoneural region (arrowhead). (d) Higher magnification image of the boxed region on the right side of panel a, showing punctate staining of axonal processes in the neuropile of the ectoneural region. Absence of staining in the hyponeural region is also seen clearly here. (e) Higher magnification image of the boxed region in panel b, showing staining in the ectoneural neuropile. Note that there is a band of strongly stained large fibers at the boundary between the largely unstained oral region of neuropile and the immunostained aboral region of neuropile (bracket). Note also the columnar organization of the neuropile, with columns of stained axonal processes separated by the unstained processes of supporting cells (radial glial‐like cells; arrow). (f) AruRGP‐immunoreactivity in the marginal nerve. (g) Parasagittal section of a radial nerve cord showing AruRGP‐immunoreactivity in the ectoneural region, but not in hyponeural region. Note also the presence of an immunostained cell (arrowhead) in the ectoneural epithelium. (h) Higher magnification image of the boxed region in panel g. Note the banded appearance of the immunostaining, with two clusters of longitudinally projecting immunostained axons (asterisks) that are separated and bounded by three less intensely stained regions of neuropile (triangles). (i) Horizontal section of a juvenile starfish showing AruRGP‐immunoreactive fibers in the ectoneural neuropile at the junction between a radial nerve cord and the circumoral nerve ring. (j) Transverse section of circumoral nerve ring showing AruRGP‐immunoreactivity in the ectoneural region but not in the hyponeural region, which has become detached from the ectoneural region during processing of this tissue section. AruRGP‐immunoreactivity in the circumoral nerve ring is continuous with immunostaining in the subepithelial nerve plexus of the peristomial membrane. (k) Higher magnification image of lower boxed region in panel j, showing stained axonal processes in the ectoneural neuropile proximal to where the hyponeural region is located naturally, but which has become detached during tissue processing (as seen in panel j). (l) Higher magnification image of the upper boxed region of panel j showing intense AruRGP‐immunoreactivity at the junction between the circumoral nerve ring and the peristomial membrane. BW, body wall; CONR, circumoral nerve ring; Ec, ectoneural region; Hy, hyponeural region; MN, marginal nerve; OHR, oral hemal ring; PM, peristomial membrane; RHS, radial hemal strand or sinus; RNC, radial nerve cord; TF, tube foot. Scale bar: 40 μm in panels a, b, f, g, i, and j; 10 μm in panels c, d, e, h, k, and l.

Immunohistochemical analysis of transverse sections of arms using the AruRGP antiserum revealed immunostaining in the ectoneural region of the radial nerve cords (Figure 5a–e; see also Figure 1b,c) and in the marginal nerves (Figure 5f; see also Figure 1c), and the specificity of this staining was confirmed by absence of staining in arm sections incubated with AruRGP antiserum preabsorbed with synthetic AruRGP (Figure 5a, inset; Figure S1f). No immunostaining was observed in the hyponeural region of the radial nerve cords. In transverse sections of the radial nerve cords, clusters of two to three immunostained cells were revealed in the ectoneural epithelium, located midway between the apex of the nerve cord and the junction of the nerve cord with adjacent tube feet. Accordingly, a bilaterally symmetrical pattern of AruRGP immunoreactivity was observed in neuropile of the ectoneural region, with immunostained fibers concentrated in the midsection of the two arms of the V‐shaped nerve cord and with little or no staining present laterally or at the apex of the V (Figure 5a,b). In transverse sections of the arm near to the arm tip, a uniform pattern of punctate immunostained fibers was observed in the ectoneural neuropile (Figure 5a,c,d). However, in transverse sections proximal to the junction of the radial nerve with the circumoral nerve ring, a distinct band of larger stained fibers can be observed at the boundary between the aboral part of the ectoneural neuropile, which contains uniformly distributed finer immunostained processes (Figure 5b,e), and the adjacent oral part of the ectoneural neuropile, which is largely void of immunostaining (Figure 5a,b). Accordingly, in sagittal sections of arms, a banded pattern of immunolabeling is observed in the radial nerve cords, with strongly stained fibers running longitudinally. In sagittal sections, sparsely distributed immunostained cells can be observed in the ectoneural epithelium of the radial nerve cords (Figure 5g,h). In horizontal sections of juvenile *A. rubens*, immunostained fibers can be observed in the ectoneural neuropile projecting between the radial nerve cords and the circumoral nerve ring but without crossing the midline of the radial nerve cords (Figure 5i). In transverse sections of the central disc, AruRGP‐immunoreactive fibers can be observed in the ectoneural region of the circumoral nerve ring located proximal to the hyponeural region, which is unstained (Figure 5j,k; see also Figure 1b). Furthermore, the immunostaining in the ectoneural region of the circumoral nerve ring can be seen to be contiguous with immunostaining in the subepithelial nerve plexus of the adjacent peristomial membrane that surrounds the mouth (Figure 5l).

#### 3.2.2 Tube feet, arm tip, and body wall

**FIGURE 6:**
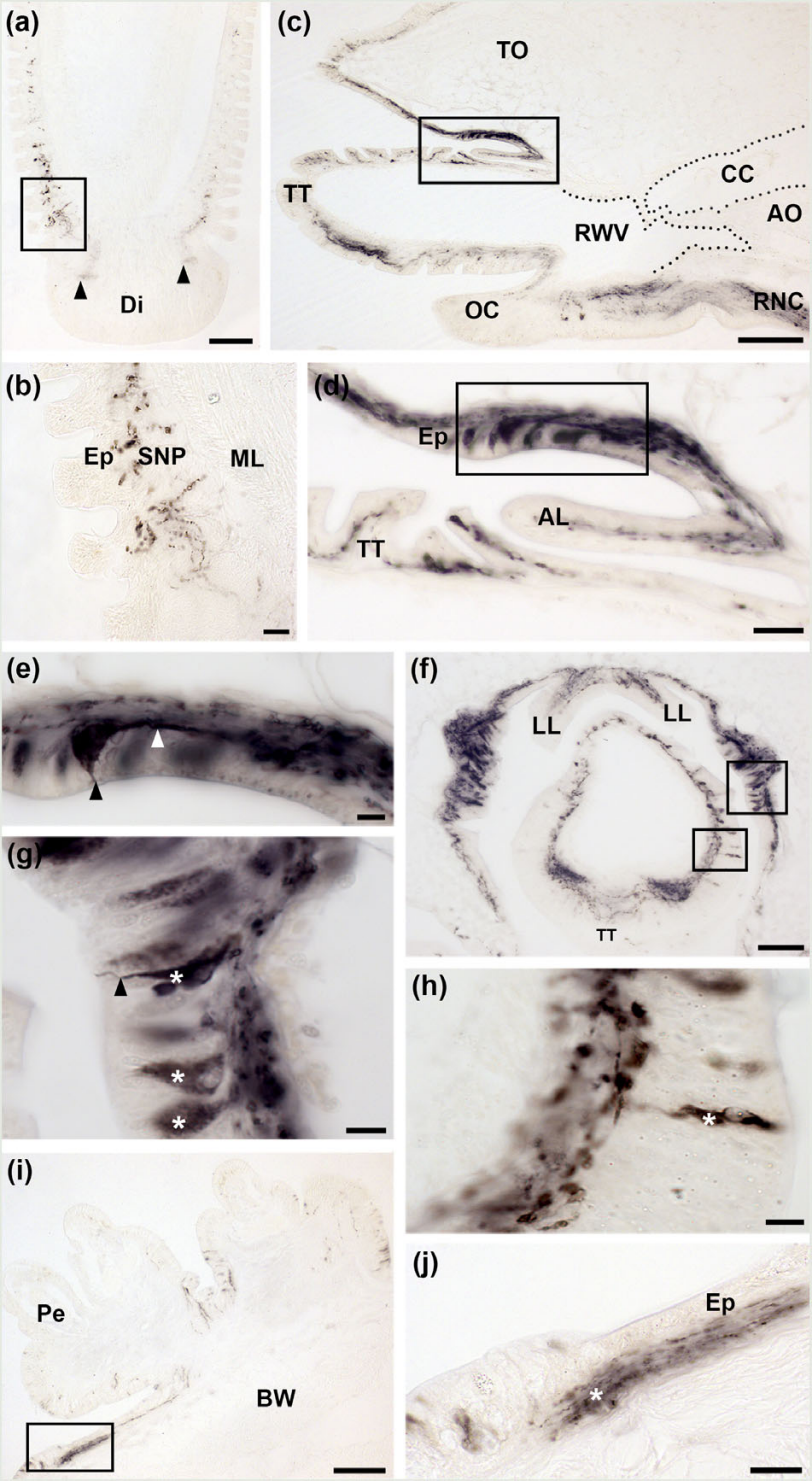
Immunohistochemical localization of AruRGP in tube feet, arm tip, and body wall of *Asterias rubens*. (a) Longitudinal section of a tube foot showing AruRGP‐immunoreactivity in the subepithelial nerve plexus of the podium (boxed region) and in the basal nerve ring (arrowheads) proximal to the disc region. (b) Higher magnification image of boxed region in panel a, showing immunostained fibers in the subepithelial nerve plexus. (c) Sagittal section of an arm tip showing AruRGP‐immunoreactivity in the body wall subepithelial nerve plexus, terminal tentacle, optic cushion, and radial nerve cord. The margins of the coelomic cavity and radial water vessel of the terminal tentacle are labeled with dotted lines to highlight the presence of tissue that separates the coelomic cavity and radial water vessel. (d) Higher magnification image of boxed region in panel c, showing immunostained cells and/or processes in the aboral body wall epithelium above the terminal tentacle, in the aboral lappet, and in the terminal tentacle. (e) Higher magnification image of boxed region in panel d, showing a strongly immunostained bipolar shaped cell body in the body wall epithelium, with a stained neurite projecting toward the external surface of the epithelium (black arrowhead) and a stained axonal process projecting into the subepithelial nerve plexus (white arrowhead). (f) Transverse section of an arm tip showing AruRGP‐immunoreactivity in the terminal tentacle, lateral lappet, and body wall epithelium surrounding the cavity containing the terminal tentacle. (g) Higher magnification image of the right boxed region of panel f showing immunostained cells (asterisks) in the body wall epithelium. A stained neurite of one of the cells can be seen projecting to the external surface of the epithelium (black arrowhead). (h) Higher magnification image of left boxed region of panel f showing a bipolar shaped immunostained cell in the epithelial layer of the terminal tentacle (asterisk) with an axonal process projecting into the underlying subepithelial nerve plexus. (i) AruRGP‐immunoreactivity in the aboral body wall epithelium and subepithelial nerve plexus of an arm, proximal to a cluster of pedicellariae. (j) Higher magnification image of the boxed region in panel i showing AruRGP‐immunoreactivity in the subepithelial nerve plexus (asterisk). AL, aboral lappet; AO, ambulacral ossicles; BNR, basal nerve ring; BW, body wall; CC, coelomic cavity; Di, disk; Ep, epithelium; LL, lateral lappet; ML, muscle layer; OC, optic cushion; Pe, pedicellaria; RWV, radial water vessel; SNP, subepithelial nerve plexus; TO, terminal ossicle; TT, terminal tentacle. Scale bar: 100 μm in panels a, b, and i; 50 μm in panel e; 20 μm in panels c, d, g, and h; 6 μm in panels f and j.

Immunostained cells and/or fibers were revealed in the subepithelial nerve plexus and basal nerve ring of tube feet (Figure 6a,b). Sagittal (Figure 6c–e; see also Figure 1d) and transverse (Figure 6f–h) sections of arm tips revealed extensive immunostaining, with stained cells and/or fibers present in the optic cushion, the terminal tentacle, the aboral (Figure 6c,d) and lateral (Figure 6f) lappets, and the body wall epithelium that lines the cavity containing the terminal tentacle (Figure 6c,f). Immunolabeling in the optic cushion is contiguous with the immunostained ectoneural neuropile of the adjacent distal region of the radial nerve cord (Figure 6c). In the terminal tentacle, immunostained bipolar shaped cells are located in the external epithelial layer (Figure 6h), with their immunostained axonal processes contributing to the immunolabeled subepithelial nerve plexus, which is thickened on the oral side of the terminal tentacle (Figure 6f,h). High‐magnification images of the body wall epithelium surrounding the cavity that contains the terminal tentacle revealed intensely stained bipolar shaped neuronal cell bodies, with an apical process projecting to the surface of the body wall epithelium and a thick axonal process located basally (Figure 6e). However, none of the immunostained processes originating in the arm tip were observed to project into or to terminate proximal to the coelomic cavity of the arm (Figure 6c).

Consistent with the presence of immunostaining in the external epithelium of the arm tips, AruRGP‐immunoreactivity was also observed in the external epithelium of the body wall in other regions of the arm, but with the density and intensity of immunostained processes being lower than in the arm tip. Thus, immunostained cells and processes can be seen in close association with pedicellariae, pincer‐like body wall appendages located on the aboral surface that keep it clear of debris (Figure 6i,j; see also Figure 1b,c).

#### 3.2.3 Digestive system

**FIGURE 7:**
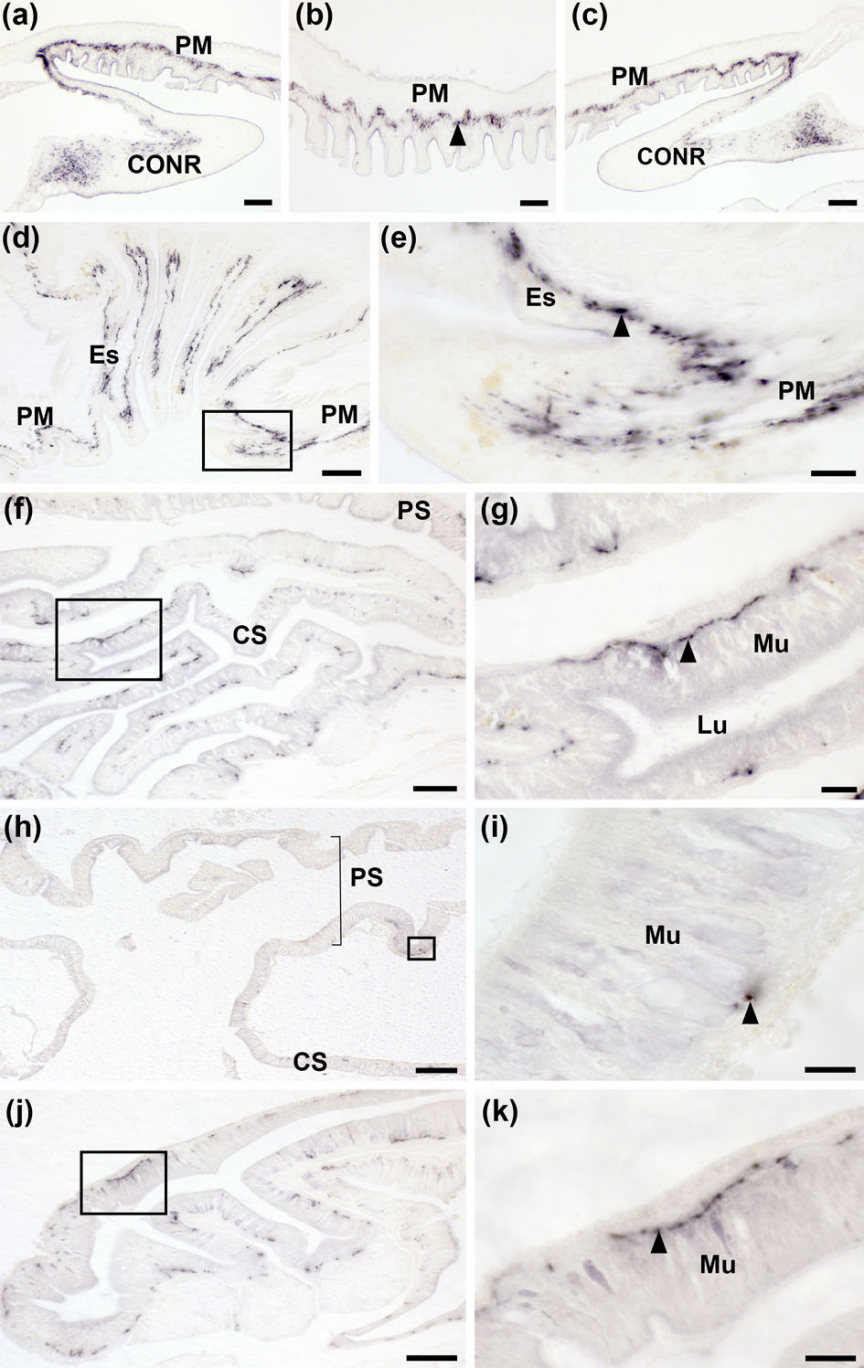
Immunohistochemical localization of AruRGP in the digestive system of *Asterias rubens*. (a–c) Transverse section of the central disc region showing AruRGP‐immunoreactivity in the subepithelial nerve plexus of the peristomial membrane, which is contiguous with immunostaining in the adjacent ectoneural region of the circumoral nerve ring. (d) Transverse section of a central disc showing AruRGP‐immunoreactivity in the subepithelial nerve plexus of the peristomial membrane and esophagus. (e) Higher magnification image of the boxed region of panel d showing immunostained fibers in the subepithelial nerve plexus at the junction between the peristomial membrane and the esophagus. (f) Transverse section of a central disc showing AruRGP‐immunoreactivity in the highly folded cardiac stomach. (g) Higher magnification image of the boxed region in panel f showing AruRGP‐immunoreactivity in the basiepithelial nerve plexus (arrowhead) of cardiac stomach. (h) Transverse section of a central disc showing sparse AruRGP‐immunoreactivity in the pyloric stomach. (i) Higher magnification image of boxed region in panel h showing immunostaining in the basiepithelial nerve plexus (arrowhead). (j) AruRGP‐immunoreactivity in a transverse section of a pyloric caecum. (k) Higher magnification image of the boxed region in panel j showing immunostaining in the basiepithelial nerve plexus (arrowhead). CONR, circumoral nerve ring; CS, cardiac stomach; Es, esophagus; Lu, lumen of gut; Mu, mucosa; PM, peristomial membrane; PS, pyloric stomach. Scale bar: 200 μm in panel h; 80 μm in panels d, f, and j; 60 μm in panels a–c; 20 μm in panels e and i; 10 μm in panels i and k.

AruRGP‐immunoreactivity was revealed in several regions of the digestive system (refer to Figure 1b,c for anatomy), including the peristomial membrane (Figure 7a–c), esophagus (Figure 7d,e), cardiac stomach (Figure 7f,g), pyloric stomach (Figure 7h,i), and pyloric caeca (Figure 7j,k). AruRGP‐immunoreactivity was localized in the basiepithelial nerve plexus in each of these regions of the digestive system. The AruRGP‐immunoreactivity in the peristomial membrane was continuous with immunostaining in the ectoneural region of the circumoral nerve ring (Figure 7a–c). AruRGP‐immunoreactivity is also present in the basiepithelial nerve plexus of the esophagus (Figure 7d), and a high‐magnification image of immunostaining at the junction of the peristomial membrane and the esophagus is shown in Figure 7e. Extensive AruRGP‐immunoreactivity was revealed in the basiepithelial nerve plexus of the cardiac stomach (Figure 7f,g), but little staining was observed in the pyloric stomach (Figure 7h,i). Lastly, AruRGP‐immunoreactivity was also revealed in the basiepithelial nerve plexus of the pyloric caeca (Figure 7j,k).

#### 3.2.4 Reproductive system

**FIGURE 8:**
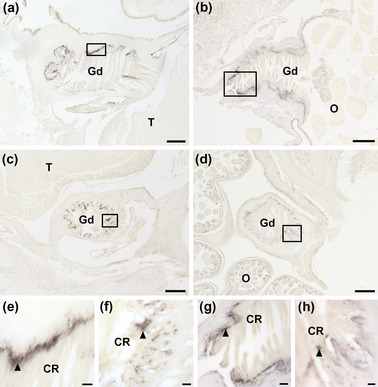
Immunohistochemical localization of AruRGP in the reproductive system of *Asterias rubens*. (a–d) AruRGP‐immunoreactivity in longitudinal (a, b) and transverse (c, d) sections of the gonoduct in male (a, c) and female (b, d) starfish. Note the absence of immunostaining in the testes (a, c) and ovaries (O). (e) Higher magnification image of the boxed region in panel a showing AruRGP‐immunoreactivity in a nerve plexus (arrowhead) located at the base of the cross‐ridged luminal epithelial layer of the gonoduct (male). (f) Higher magnification image of the boxed region in panel c showing AruRGP‐immunoreactivity in a nerve plexus (arrowhead) located at the base of the cross‐ridged luminal epithelial layer of the gonoduct (male). (g) Higher magnification image of the boxed region in panel b showing AruRGP‐immunoreactivity in a nerve plexus (arrowhead) located at the base of the cross‐ridged luminal epithelial layer of the gonoduct (female). (f) Higher magnification image of the boxed region in panel d showing AruRGP‐immunoreactivity in a nerve plexus (arrowhead) located at the base of the cross‐ridged luminal epithelial layer of the gonoduct (female). CR, cross‐ridge epithelium; Gd, gonoduct; O, ovary; T, testis. Scale bar: 150 μm in panels a–d; 20 μm in panels e–h.

No immunostaining was observed in testes (Figure 8a,c) or ovaries (Figure 8b,d), but AruRGP‐immunoreactivity was revealed in the gonoducts (Figure 8a–d; see also Figure 1b). Thus, in longitudinal and transverse sections of the gonoduct in both male and female animals, AruRGP‐immunoreactivity is localized in a basiepithelial nerve plexus located beneath the epithelium that forms the lining of the lumen of the gonoduct (Figure 8e–h).

### 3.3 In vitro pharmacology

No myoexcitatory (muscle contraction) or myoinhibitory (muscle relaxation) effects of AruRGP on cardiac stomach or tube feet preparations were observed (Figure S2).

## 4 DISCUSSION

The molecular identification of RGP as a gonadotropin in starfish (Mita, Yoshikuni, et al., 2009) has provided a basis for important advances in our knowledge and understanding of the reproductive neuroendocrinology of echinoderms. For example, it has been discovered that an RGP‐like peptide also triggers spawning in sea cucumbers, demonstrating that this role of relaxin‐type peptides is not unique to starfish (Chieu et al., 2019). However, there is still much to be learnt about the physiological mechanisms by which RGP acts as regulator of spawning in starfish and in other echinoderms.

Because RGP was originally isolated from extracts of starfish radial nerve cords, it has generally been assumed that the radial nerve cords are the physiological source of RGP that triggers spawning in starfish (Mita, 2019). However, as discussed in more detail below, there is no direct evidence in support of this hypothesis. Furthermore, prior to the molecular identification of RGP, it was already known that extracts of other tissues/organs contain GSS/RGP‐like bioactivity, including tube feet, cardiac stomach, and body wall of *A. amurensis*, albeit at much lower concentrations than in the radial nerve cords (Kanatani & Ohguri, 1966). These findings have been confirmed following the molecular characterization of RGP, as summarized in Table 1. Thus, using PCR and/or immunoassay methods, RGP expression has been detected in the radial nerve cords, circumoral nerve ring, cardiac stomach, pyloric caeca, pyloric stomach, and tube feet of *P. pectinifera* (Haraguchi et al., 2016; Mita & Katayama, 2018; Mita, Ito, et al., 2009; Mita, Yoshikuni, et al., 2009; Yamamoto et al., 2017). However, a limitation of these biochemical detection methods is their lack of resolution anatomically. Therefore, in this study, both mRNA in situ hybridization and immunohistochemistry were used to enable the first comprehensive analysis of the expression of RGP in starfish, using the common European species *A. rubens* as an experimental model and as summarized in Table 1. Below, we discuss the physiological significance of our findings and, in particular, how they provide insights into the mechanisms by which RGP acts as a regulator of spawning in starfish.

**TABLE 1:** Summary of evidence of GSS/RGP expression in tissues/organs of several starfish species using a variety of experimental techniques.

		Evidence of GSS/RGP expression in starfish organs/tissues														
Species	Technique	RNC	CONR	MN	PM	E	CS	PS	PC	TF	TT	BW	G	GD	C	References
*Asterias rubens*	IHC	*	*	*	*	*	*	*	*	*	*	*	nd	*	ni	This study
	ELISA	*	*	ni	ni	ni	*	*	nd	*	ni	ni	nd	ni	ni	Mita, Elphick, et al., 2021
	MS	*	ni	ni	ni	ni	ni	ni	ni	ni	ni	ni	ni	ni	ni	Lin, Mita, et al., 2017 and this study
	ISH	*	*	nd	nd	nd	*	*	nd	*	*	nd	nd	*	ni	
*Asterias amurensis*	Bioassay	*	*	ni	ni	ni	*	ni	ni	ni	ni	*	ni	ni	ni	Kanatani, 1964; Kanatani & Ohguri, 1966
*Asterias forbesi*	Bioassay	*	ni	ni	ni	ni	ni	ni	ni	ni	ni	ni	ni	ni	ni	Chaet & McConnaughy, 1959
*Patiria (Asterina) pectinifera*	IHC	*	ni	ni	ni	ni	ni	ni	ni	ni	ni	ni	ni	ni	ni	Yamamoto et al., 2017
	ELISA	*	*	ni	ni	ni	nd	nd	ni	ni	ni	ni	nd	ni	ni	Mita & Katayama, 2018
	MS	*	ni	ni	ni	ni	ni	ni	ni	ni	ni	ni	ni	ni	ni	Mita, Yoshikuni, et al., 2009
	RIA	*	*	ni	ni	ni	nd	nd	nd	nd	ni	ni	nd	ni	ni	Yamamoto et al., 2017
	Bioassay	*	*	ni	ni	ni	*	ni	nd	*	ni	*	nd	ni	ni	Kanatani & Ohguri, 1966; Mita, Ito, et al., 2009
	ISH	*	ni	ni	ni	ni	ni	ni	ni	ni	ni	ni	ni	ni	ni	Mita, Yoshikuni, et al., 2009
	PCR	*	ni	ni	ni	ni	nd	ni	nd	nd	ni	ni	nd	ni	ni	Mita, Ito, et al., 2009
	RT‐qPCR	*	ni	ni	ni	ni	*	ni	*	*	ni	ni	*	ni	ni	Haraguchi et al., 2016
*Acanthaster* cf. *solaris*/*Acanthaster planci*	IHC	*	ni	ni	ni	ni	ni	ni	ni	ni	ni	ni	ni	ni	ni	Mita et al., 2022
	ELISA	*	ni	ni	ni	ni	ni	ni	ni	ni	ni	ni	nd	ni	ni	
	MS	*	ni	ni	ni	ni	ni	ni	ni	*	ni	*	*	ni	ni	Smith et al., 2017
	Bioassay	*	ni	ni	ni	ni	*	ni	nd	*	ni	ni	nd	ni	ni	Mita et al., 2022
	RNA‐seq	*	ni	ni	ni	ni	ni	ni	ni	ni	*	*	*	ni	*	Jönsson et al., 2022

Abbreviations: *, detected; BW, body wall; C, coelomocytes; CONR, circumoral nerve ring; CS, cardiac stomach; E, esophagus; ELISA, enzyme‐linked immunosorbent assay; G, gonad; GD, gonoduct; IHC, immunohistochemistry; ISH, mRNA in situ hybridization; MN, marginal nerve; MS, mass spectrometry; nd, not detected; ni, not investigated; PCR, polymerase chain reaction; PM, peristomial membrane; PS, pyloric stomach; RIA, radioimmunoassay; RNA‐seq, transcriptomic RNA sequencing; RNC, radial nerve cord; RT‐qPCR, real‐time quantitative PCR; TF, tube foot; TT, terminal tentacle.

### 4.1 Functional significance of RGP expression in the central nervous system

The distribution of cells expressing RGP precursor transcripts in the radial nerve cords has been reported previously in *A. rubens* (Lin, Mita, et al., 2017) and in *P. pectinifera* (Mita, Yoshikuni, et al., 2009). It is noteworthy that, by comparison with many other neuropeptide types, the relative abundance of cells expressing RGP is quite low. Thus, many other neuropeptide types are expressed in both the ectoneural and the hyponeural regions of the radial nerve cords in *A. rubens* (Cai et al., 2023, 2018; Lin et al., 2018; Lin, Egertova, et al., 2017; Tian et al., 2017; Tinoco et al., 2021; Zhang et al., 2020, 2022), whereas expression of RGP is restricted to the ectoneural region. Furthermore, the number of cells expressing RGP in the ectoneural region is quite low, with bilaterally symmetrical clusters of only two to three stained cells revealed in transverse sections of radial nerve cords. This contrasts with many other neuropeptide types (e.g., ArCT, ArCRH, ArPPLN1b, ArPPLN2h) where larger numbers of stained cells are revealed in transverse sections of radial nerve cords (Cai et al., 2023, 2018; Cobb, 1985; Lin et al., 2018; Lin, Egertova, et al., 2017). The functional significance of differences in the relative abundance of cells expressing different neuropeptides in the ectoneural region of radial nerve cords is not known. However, use of immunohistochemistry has provided new insights into RGP expression in the radial nerve cords because, unlike with mRNA in situ hybridization, the stained cells and processes (axons) of RGP‐expressing neurons are revealed. Previous studies have reported detection of RGP‐immunoreactivity in cells and neuropile of the ectoneural region (Yamamoto et al., 2017). Here, our immunohistochemical analysis of RGP expression in *A. rubens* revealed a distinct population of large nerve fibers expressing RGP that project longitudinally through the neuropile of the ectoneural region. The functional significance of these RGP‐expressing fibers is not known, but it suggests that RGP signaling may be involved in intersegmental neuronal communication. Furthermore, the absence of RGP expression in the hyponeural region is noteworthy because it is this region of the central nervous system that contains the cell bodies of motoneurons that co‐ordinate whole‐animal behavior by controlling the activity of tube feet and interossicular muscles of the body wall in starfish. The dendrites of hyponeural motoneurons project into and receive presynaptic input from the ectoneural region (Cobb, 1985; Mashanov et al., 2016; Smith, 1937; Zueva et al., 2018) and therefore it is possible that RGP released within the ectoneural neuropile influences whole‐animal behavior by modulating the activity of hyponeural neurons. Accordingly, it is noteworthy that in the circumoral nerve ring, RGP‐expressing fibers were found to be concentrated in the innermost layer of the ectoneural neuropile, proximal to the hyponeural region.

Prior to the molecular identification of GSS as RGP, it was proposed that GSS is synthesized in supporting cells (radial glia) in the ectoneural region of the radial nerve cord and then transported through supporting fibers to the radial and transverse hemal sinus of the hemal system, via which GSS could in theory reach the gonads (Mita, 2019; Unger, 1962). Now, with the specific visualization RGP in the ectoneural region of the radial nerve cord reported here, it is possible to evaluate this hypothesis neuroanatomically. Our findings show that RGP is not expressed in supporting cells (radial glia) but instead RGP is expressed in neuronal cells with extensive axonal processes that project longitudinally in the ectoneural region of the radial nerve cords. Furthermore, no evidence of RGP‐expressing fibers projecting from the ectoneural region to the radial and transverse hemal sinus was observed. Another potential route by which RGP could in theory travel to the gonads from the radial nerve cords is via the perihemal coelomic spaces. However, it seems unlikely that RGP released by neuronal fibers in the ectoneural region could gain access to the perihemal coelom because the hyponeural region separates it from the ectoneural region. Based on these anatomical considerations, we suggest that it is unlikely that the ectoneural region of the radial nerve cords and/or circumoral nerve ring is the physiological source of RGP that triggers gamete maturation and spawning in starfish. However, spawning in starfish involves more than just the release of gametes into the surrounding seawater because it is also accompanied by changes in whole‐animal behavior. Thus, starfish typically adopt a humped posture when spawning, with animals standing on the tips of their arms and thereby bringing the sites of gamete release (gonopores) off the seabed into the water column above, which may facilitate gamete dispersal (Minchin, 1987). It is possible, therefore, that RGP‐expressing neurons in the ectoneural region of the radial nerve cords participate in neural mechanisms underlying the adoption and maintenance of this spawning posture.

### 4.2 Functional significance of RGP expression in the tube feet, arm tips, and body wall

We have reported previously the expression of RGP precursor transcripts in the tube feet, arm tips, and associated sensory organs of *A. rubens* (Lin, Mita, et al., 2017). By using immunohistochemistry, here we have obtained more detailed insights into the neuroanatomy of RGP‐expressing cells in these regions of the starfish body because their axonal processes are visualized using this technique. Thus, in the tube feet, RGP‐immunoreactive fibers are revealed in the subepithelial nerve plexus and basal nerve ring. Informed by this pattern of expression, we investigated if RGP affects the contractile state of tube foot preparations in vitro, but we found that it neither causes contraction nor relaxation of tube feet when tested at 10^−6^ M.

An extensive system of RGP‐expressing cells/fibers has been revealed in the arm tips, with the architecture of immunostained cells and their processes revealed in the terminal tentacle (a mechanosensory organ), the optic cushion (a photosensory organ), the lateral and aboral lappets (presumptive chemosensory organs), and more generally in the epithelium of the body wall cavity that surrounds the terminal tentacle. Because of the extensive expression of RGP in the arm tip and its associated sensory organs, we have proposed previously that the arm tips may be the physiological source of RGP that triggers spawning in starfish, with the rationale being that RGP‐expressing cells in the arm tip and associated sensory organs could integrate sensation of changes in environmental conditions (e.g., day length, lunar cycle, pheromones) thought to trigger spawning (Lin, Mita, et al., 2017). However, if arm tips are the source of RGP that triggers spawning, RGP released by cells in the arm tips would need to gain access to coelomic cavity of the arms where the gonads are located. Because in situ hybridization typically only enables visualization of transcripts in cell bodies, our previous analysis of RGP expression did not enable us to address this issue. Now with the availability of specific antibodies to AruRGP and use of immunohistochemistry, importantly, we did not observe any evidence of the axonal processes of RGP‐expressing cells in the arm tips terminating proximal to the coelomic cavity. Therefore, as with RGP‐expressing cells in the radial nerve cords and circumoral nerve ring, we conclude that the RGP‐expressing cells in the arm tips are unlikely to be the physiological source of RGP that triggers spawning in starfish. However, the axonal processes of RGP‐expressing cells in the arm tips may contribute to immunostaining observed in the ectoneural neuropile of the radial nerve cords because there is continuity with the neuropile regions of the terminal tentacle and optic cushion. Thus, while the RGP‐expressing cells in the arm tip may not be directly involved in triggering gamete maturation and release, it is possible they are involved in neural mechanisms that initiate and maintain the humped posture that starfish adopt when they spawn.

Another indication that RGP expression in the arm tips may not be specifically associated with physiological mechanisms of RGP‐induced gamete maturation and release is our discovery that RGP‐immunoreactivity is detected not only in the body wall of the arm tips but also in the body wall in other regions of the arms.

### 4.3 Functional significance of RGP expression in the digestive system

The expression of RGP in the cardiac stomach of starfish has been reported previously based on analysis of tissue extracts for GSS/RGP bioactivity, PCR‐based detection of RGP transcripts, or immunoassays (Haraguchi et al., 2016; Mita & Katayama, 2018, 2022; Mita, Ito, et al., 2009). However, this is the first study to visualize RGP expression in the starfish digestive system using mRNA in situ hybridization and immunohistochemistry. Cells expressing AruRGP precursor transcripts were visualized in the cardiac stomach and pyloric stomach of *A. rubens*, but these were sparse in number by comparison with many other neuropeptides we have analyzed previously (Cai et al., 2023, 2018; Lin et al., 2018; Lin, Egertova, et al., 2017; Tian et al., 2017; Tinoco et al., 2021; Zhang et al., 2020, 2022). Nevertheless, use of AruRGP antibodies enabled visualization of the axonal processes of these cells, revealing that they contribute extensive immunostaining in the basiepithelial nerve plexus of the cardiac stomach. Furthermore, RGP‐immunoreactivity was also observed in other regions of the digestive system in *A. rubens*, including the peristomial membrane that surrounds mouth and the esophagus that links the mouth and the cardiac stomach. Additionally, although cells expressing RGP were not revealed in the pyloric caeca by mRNA in situ hybridization, immunostaining was observed in the basiepithelial nerve plexus of these digestive organs. Collectively, these findings indicate that RGP may be involved in regulation of feeding and/or digestive physiology in starfish.

Our previous investigations have revealed that several neuropeptides expressed in the cardiac stomach of *A. rubens* are myoactive, causing relaxation or contraction of in vitro preparations of this organ. This is of interest with respect to the extraoral feeding behavior of starfish, where in species such as *A. rubens* the cardiac stomach is everted out of the mouth over the soft tissues of prey (e.g., mussels) and then is retracted when partial digestion and uptake of prey tissues are completed (Anderson, 1954; Lawrence, 2013). For example, the vasopressin/oxytocin‐type neuropeptide asterotocin causes cardiac stomach relaxation in vitro and eversion in vivo (Odekunle et al., 2019) and the neuropeptide NGFFYamide causes cardiac stomach contraction in vitro and retraction in vivo (Semmens et al., 2013; Tinoco et al., 2018). Because AruRGP is expressed in the cardiac stomach of *A. rubens*, here we investigated if it has in vitro pharmacological effects on this organ and found that it causes neither relaxation nor contraction of cardiac stomach preparations. Therefore, AruRGP is presumably involved in regulation of other physiological processes in the digestive system of *A. rubens*.

### 4.4 Functional significance of RGP expression in the gonoducts

Previous studies, using bioassays, radioimmunoassays, or ELISAs, have investigated if RGP is expressed in starfish gonads and these have typically reported that RGP expression is not detected (Table 1). Accordingly, our analysis of *A. rubens* of using mRNA in situ hybridization and immunohistochemistry revealed no evidence of RGP expression in gonads. However, we have for the first time discovered that RGP is expressed in the gonoducts, tubular shaped structures that link the gonads to the gonopores. The small size and inaccessibility of the gonoducts probably explain why previous investigations using biochemical assays have not investigated or detected RGP expression in these organs.

**FIGURE 9:**
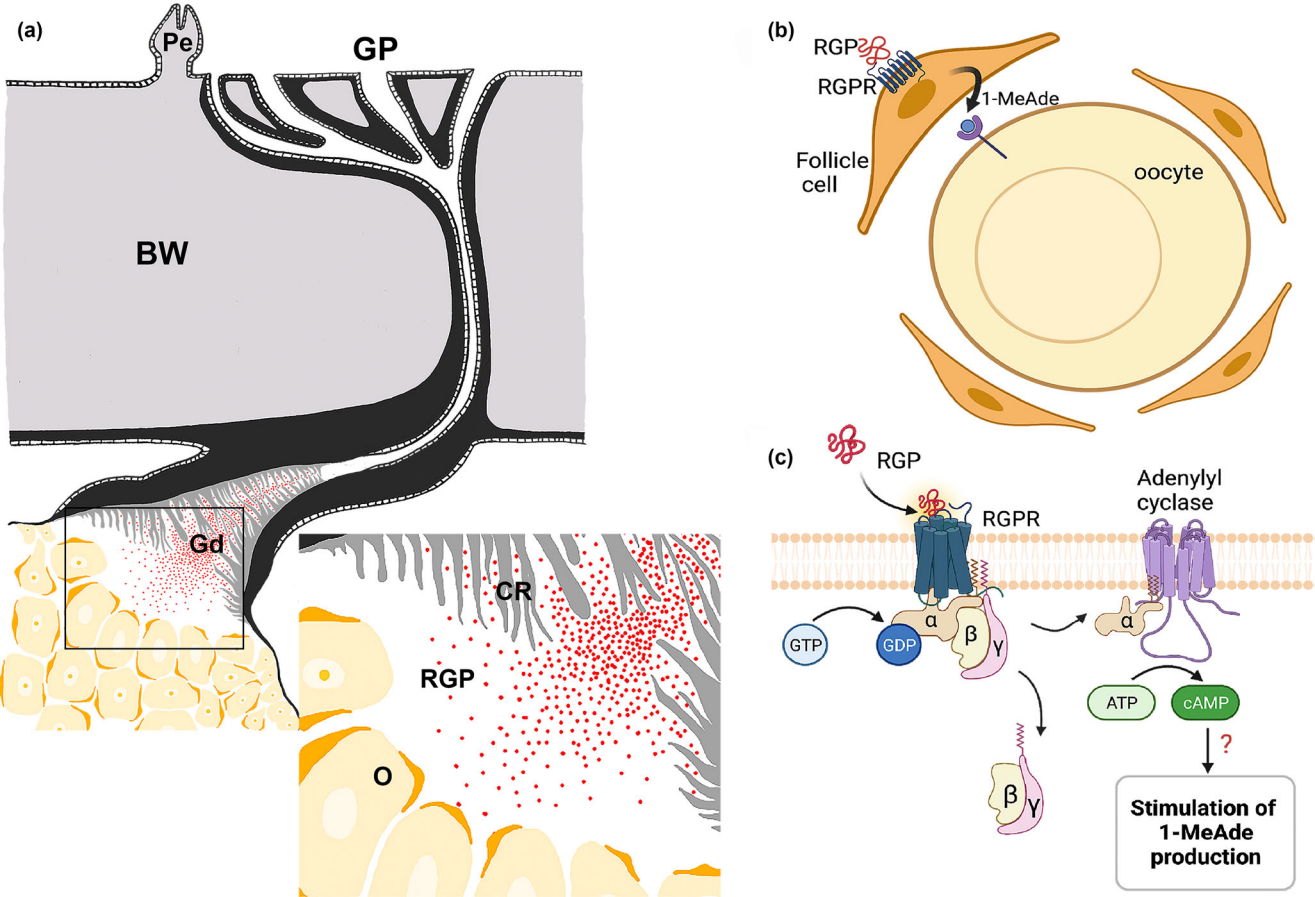
Schematic diagrams showing the anatomy of RGP signaling in starfish, with the gonoduct as a source of RGP and the gonad as a site of action. (a) Diagram showing a model of our hypothesis that prior to spawning in starfish, RGP molecules released from nerve processes in the gonoduct diffuse into lumen of the gonoduct and then into the lumen of the adjoining gonad (ovary), where they can bind to cell surface receptor proteins on follicle cells surrounding immature oocytes. (b) Diagram showing how binding of RGP to RGP receptors on follicle cells stimulates formation of 1‐methyladenine, which then binds to receptors on oocytes to trigger oocyte maturation (germinal vesicle breakdown). (c) Detail of signal transduction pathway in follicle cells, in which binding of RGP to a G‐protein‐coupled receptor triggers G‐protein (G_s_)‐mediated stimulation of adenylyl cyclase activity and elevation of intracellular cyclic adenosine monophosphate, which then triggers formation of 1‐methyladenine via as yet unknown molecular mechanisms. α, alpha subunit of G‐protein; ATP, adenosine triphosphate; β, beta subunit of G‐protein; BW, body wall; Cr, cross‐ridged epithelium of gonoduct; γ, gamma subunit of G‐protein; Gd, gonoduct; GDP, guanosine diphosphate; GTP, guanosine triphosphate; 1‐MeAde, 1‐methyladenine; O, ovary; RGP, relaxin‐like gonad‐stimulating peptide; RGPR, RGP receptor. The diagram in panel a is adapted from figure 1 in Walker (1975) and was drawn in Photoshop CC2020 (San Jose, CA) using the digital pen of a creative drawing tablet (Wacom, Tokyo, Japan), which was connected to a laptop (MacBook Pro, Apple, USA). The diagrams in panels b and c are based upon figure 2 in Mita (2019) and were created using BioRender (https://www.biorender.com/).

Detailed descriptions of the anatomy of the gonoducts in *Asterias* have been reported previously (Walker, 1975). A distinctive feature of the gonoducts is the presence of cross‐ridges in the luminal epithelium that occlude the lumen and are thought to act as a barrier to prevent gametes from exiting the gonad to the gonopore prior to spawning. Interestingly, use of in situ hybridization revealed AruRGP precursor transcripts in cells located basal to the luminal epithelium at a position proximal to the junction of the gonoduct with the gonopore. Furthermore, immunohistochemical analysis of AruRGP expression revealed extensive immunoreactivity in the basiepithelial nerve plexus located beneath the luminal epithelium of the gonoducts, in both male and female animals. These observations indicate that there is a population of RGP‐expressing neurons located in the wall of the gonoducts with axonal processes that account for immunostaining observed in the basiepithelial nerve plexus of the gonoduct. Importantly, these findings provide a new perspective on the mechanisms by which RGP acts as a regulator of spawning in starfish. Thus, because the gonoducts are located proximal to the gonads and the lumen of the gonoducts is continuous with the lumen of the gonads where immature gametes are located, the RGP‐expressing neurons in the gonoducts are excellent candidates as physiological sources of the RGP that triggers gamete maturation and release in starfish. As illustrated in Figure 9, we hypothesize that prior to spawning, RGP‐expressing neurons in the gonoducts release RGP and this diffuses as a neurohormone into the lumen of the gonoduct and then into the lumen of the gonads. Then, informed by findings from previous studies, in female animals the RGP binds to G‐protein‐coupled receptors expressed by follicle cells that surround immature oocytes, which then results in cAMP‐dependent production of 1‐methyladenine. 1‐Methyladenine then binds to 1‐methyladenine receptors located in the membrane of immature oocytes and this then triggers a downstream signaling cascade that causes germinal vesicle breakdown and oocyte maturation (Mita, 2019). A parallel pathway is thought to occur in male starfish where RGP binds to receptors on interstitial cells, which produce 1‐methyladenine that then triggers spermatocyte maturation and spawning (Kubota et al., 1977). Following gamete maturation, waves of contraction caused by the muscular wall of the gonads then expel gametes into the gonoducts, where breakdown of the cross‐ridges of the luminal lining facilitates rapid and effective expulsion of gametes to the external environment via the gonopores.

### 4.5 Evolution and comparative physiology of relaxin‐type neuropeptides as regulators of reproductive physiology

Lastly, it is of interest to reflect on the findings of this study from a comparative and evolutionary perspective, taking into account what is known about the physiological roles of relaxin‐type peptides in mammals. The hormone relaxin was first discovered as a substance present in the serum of pregnant guinea pigs or rabbits that causes relaxation of the pubic ligament of virgin guinea pigs. Subsequently, relaxin was found to be derived from corpus luteum in pigs, the placenta in rabbits, and the uterus in guinea pigs. Acting as an endocrine regulator, relaxin‐type peptides cause softening and hypertrophy of the pubic symphysis, cervix, uterus, and vagina during the second half of pregnancy, which prepares the maternal body to be ready for parturition. In male humans and rats, the prostate gland is a source of relaxin‐type peptides, and relaxin gene‐knockout mice exhibit poor growth of the reproductive tract, which is associated with reduced fertility (Bathgate et al., 2013, 2006). In the context of this well‐established evidence that the reproductive system is both a source and a site of action of relaxin‐type peptides in mammals, our discovery that the reproductive system is both a source (gonoduct) and a site of action (gonads) for RGP in starfish is noteworthy. It suggests that relaxin‐type peptide production and action in the context of reproductive physiology is an evolutionarily ancient phenomenon that can be traced back to the deuterostome common ancestor of vertebrates and echinoderms. Furthermore, with the recent discovery of a gene encoding a relaxin‐type peptide in crinoids (e.g., feather stars), which are a sister group to eleutherozoan echinoderms (e.g., starfish, brittle stars, sea urchins, sea cucumbers) (Aleotti et al., 2022), there may be opportunities to obtain further insights into the comparative physiology of relaxin‐type peptides as regulators of reproductive processes.

## AUTHOR CONTRIBUTIONS

All authors had full access to all the data in the study and take responsibility for the integrity of the data and the accuracy of the data analysis. *Study concept and design*: Yuling Feng, Masatoshi Mita, and Maurice R. Elphick. *Acquisition of data*: Yuling Feng and Michaela Egertová (mRNA in situ hybridization and immunohistochemistry); Ming Lin (production of oligonucleotide probes for AruRGPP mRNA in situ hybridization); Yuling Feng and Victor M. Piñon Gonzalez (production of gonoduct sections and immunohistochemical analysis of AruRGP expression in gonoducts); and Masatoshi Mita (production and characterization of antibodies to AruRGP). *Analysis and interpretation of data*: Yuling Feng and Maurice R. Elphick. *Drafting of the manuscript*: Yuling Feng and Maurice R. Elphick. *Obtaining sources of funding*: Maurice R. Elphick, Masatoshi Mita, Yuling Feng, and Victor M. Piñon Gonzalez. *Study supervision*: Maurice R. Elphick.

## FUNDING INFORMATION

This study was supported by China Scholarship Council studentships awarded to Y.F. and M.L., a Mexican Council of Science and Technology studentship (CONACyT studentship no. 746247) awarded to V.M.P.G., a grant awarded to M.R.E. and M.M. by the Daiwa Anglo‐Japanese Foundation, a grant awarded to M.R.E. by the Biotechnology and Biological Sciences Research Council (BBSRC; BB/M001644/1), and a grant awarded to M.M. by JSPS KAKENHI (JP19K06747).

## CONFLICT OF INTEREST STATEMENT

The authors declare no conflicts of interest.

### PEER REVIEW

The peer review history for this article is available at https://publons.com/publon/10.1002/cne.25496.

## Supporting information

Supplementary Figure 1. High magnification images for negative control experiments performed for mRNA in situ hybridization (sense probes; panels a‐e) and immunohistochemistry (pre‐absorption of antiserum with AruRGPl; panel f). (a). Inset of Figure 2a. (b). Inset of Figure 2d. (c). Inset of Figure 3a. (d). Inset of Figure 3c. (e). Inset of Figure 4a. (f). Inset of Figure 5a. Scale bars are 50 μm in a, b; 100 μm in c, d, e; 40 μm in f.

Supplementary Figure 2. AruRGP does not cause contraction or relaxation of in vitro preparations of cardiac stomach and tube feet from A. rubens. (a). Cardiac stomach preparation. AruRGP (1 μM) does not cause contraction or relaxation when tested prior to application of seawater containing 30 mM added KCl, which causes contraction. AruRGP (1 μM) also has no effect when tested after KCl‐induced contraction of the preparation. The SALMFamide neuropeptide S2 (1 μM), which acts as muscle relaxant in A. rubens (Melarange & Elphick, 2003), was tested as a positive control and caused relaxation. (b).Tube foot preparation. AruRGP (1 μM) does not cause contraction or relaxation when tested prior to application of 10 μM acetylcholine (ACh), which causes contraction. AruRGP (1 μM) also has no effect when tested after ACh‐induced contraction of the preparation.

## Data Availability

Data available on request from the authors.
