# Supplementary material for: Localization of relaxin‐like gonad‐stimulating peptide expression in starfish reveals the gonoducts as a source for its role as a regulator of spawning
Source: J Comp Neurol. 2023 May 22;531(13):1299–316. doi: 10.1002/cne.25496 (PMC10952978; doi:10.1002/cne.25496)
Supplement: Supplementary file 1 — Supplementary Figure 1. High magnification images for negative control experiments performed for mRNA in situ hybridization (sense probes; panels a‐e) and immunohistochemistry (pre‐absorption of antiserum with AruRGPl; panel f). (a). Inset of Figure 2a. (b). Inset of Figure 2d. (c). Inset of Figure 3a. (d). Inset of Figure 3c. (e). Inset of Figure 4a. (f). Inset of Figure 5a. Scale bars are 50 μm in a, b; 100 μm in c, d, e; 40 μm in f. [file CNE-531-1299-s001.pdf]

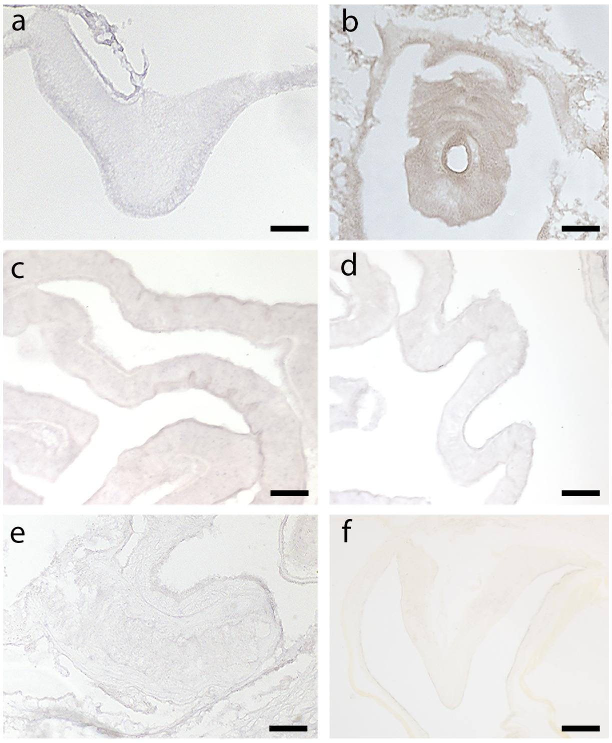

Supplementary Figure 1. High magnification images for negative control experiments performed for mRNA in situ hybridization (sense probes; panels a-e) and immunohistochemistry (pre-absorption of antiserum with AruRGPl; panel f). (a). Inset of Figure 2a. (b). Inset of Figure 2d. (c). Inset of Figure 3a. (d). Inset of Figure 3c. (e). Inset of Figure 4a. (f). Inset of Figure 5a. Scale bars are 50 μm in a, b; 100 μm in c, d, e; 40 μm in f.
