# Supplementary material for: Localization of relaxin‐like gonad‐stimulating peptide expression in starfish reveals the gonoducts as a source for its role as a regulator of spawning
Source: J Comp Neurol. 2023 May 22;531(13):1299–316. doi: 10.1002/cne.25496 (PMC10952978; doi:10.1002/cne.25496)
Supplement: Supplementary file 2 — Supplementary Figure 2. AruRGP does not cause contraction or relaxation of in vitro preparations of cardiac stomach and tube feet from A. rubens. (a). Cardiac stomach preparation. AruRGP (1 μM) does not cause contraction or relaxation when tested prior to application of seawater containing 30 mM added KCl, which causes contraction. AruRGP (1 μM) also has no effect when tested after KCl‐induced contraction of the preparation. The SALMFamide neuropeptide S2 (1 μM), which acts as muscle relaxant in A. rubens (Melarange & Elphick, 2003), was tested as a positive control and caused relaxation. (b).Tube foot preparation. AruRGP (1 μM) does not cause contraction or relaxation when tested prior to application of 10 μM acetylcholine (ACh), which causes contraction. AruRGP (1 μM) also has no effect when tested after ACh‐induced contraction of the preparation. [file CNE-531-1299-s002.pdf]

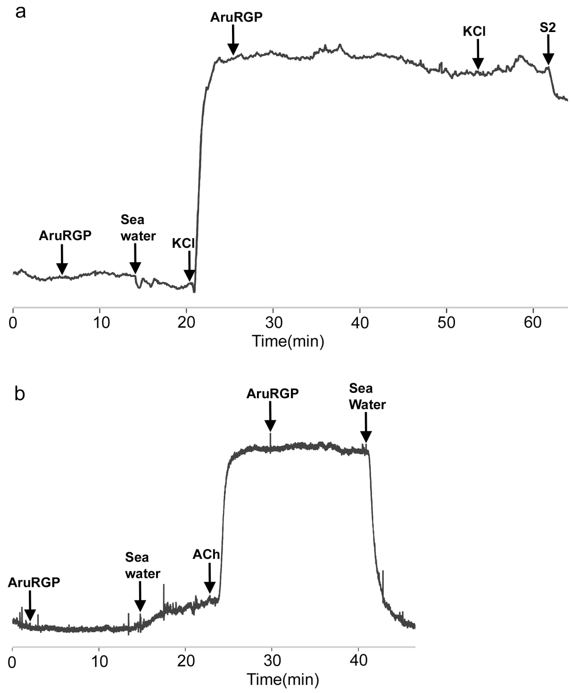

Supplementary Figure 2. AruRGP does not cause contraction or relaxation of in vitro preparations of cardiac stomach and tube feet from A. rubens. (a). Cardiac stomach preparation. AruRGP (1 μM) does not cause contraction or relaxation when tested prior to application of seawater containing 30 mM added KCl, which causes contraction. AruRGP (1 μM) also has no effect when tested after KCl-induced contraction of the preparation. The SALMFamide neuropeptide S2 (1 μM), which acts as muscle relaxant in A. rubens (Melarange & Elphick, 2003), was tested as a positive control and caused relaxation. (b).Tube foot preparation. AruRGP (1 μM) does not cause contraction or relaxation when tested prior to application of 10 μM acetylcholine (ACh), which causes contraction. AruRGP (1 μM) also has no effect when tested after ACh-induced contraction of the preparation.
